# Integrative Taxonomy to Assess the Parasitoid Complex of the Jumping Plant-Louse *Cacopsylla pulchella* (*Hemiptera*: *Psyllidae*) on *Cercis siliquastrum* in Central and Southern Italy

**DOI:** 10.3390/insects17010092

**Published:** 2026-01-13

**Authors:** Elia Russo, Gianluca Melone, Ciro Pugliese, Stefania Laudonia

**Affiliations:** 1 Department of Agricultural Sciences, University of Naples Federico II, 80055 Portici, Italy; elia.russo@unina.it (E.R.); gianluca.melone1998@gmail.com (G.M.); ciropugliese@hotmail.it (C.P.); 2 Center for Studies on Bioinspired Agro-Environmental Technology, BAT Center, University of Naples Federico II, 80055 Portici, Italy

**Keywords:** DNA barcoding, *Encyrtidae*, integrative taxonomy, *Pachyneuron aphidis*, *Pachyneuron muscarum*, parasitoid-hyperparasitoid interactions, *Prionomitus mitratus*, *Pteromalidae*, urban ecosystems

## Abstract

Urban green spaces host complex arthropod communities, in which natural insect antagonists play a key role in regulating pest populations. The jumping plant-louse *Cacopsylla pulchella* is a sap-sucking pest widespread across Europe that attacks *Cercis siliquastrum* L., which is commonly used as an ornamental tree. Heavy infestations may contribute to host tree decline and cause indirect damage in urban environments by reducing aesthetic value and by extensive deposition of honeydew secretions on surrounding surfaces. As with many phytophagous insects occurring in urban contexts, information on the natural enemies of this species remains limited, particularly in Italy, and requires further documentation. Here, we investigated the parasitoids associated with *C. pulchella* in central and southern Italy based on surveys conducted between 2022 and 2025. Specimens were obtained from infested plant material and identified using an integrative taxonomic approach combining detailed morphological examination with DNA barcoding. *Prionomitus mitratus* was confirmed as the primary parasitoid of *C. pulchella*, while two species, *Pachyneuron muscarum* and *Pachyneuron aphidis*, were identified as hyperparasitoids. In addition, a single specimen of *Anastatus bifasciatus* was also recorded emerging from the psyllid as a hyperparasitoid. Molecular analyses generated the first publicly available mitochondrial and nuclear sequences for *P. mitratus*. For *Pachyneuron*, molecular results showed variable correspondence with available reference sequences, reflecting the uneven representation of species-level data for *Pteromalidae* in public databases. By integrating morphological and molecular evidence, this study clarifies trophic relationships within the *C. pulchella* parasitoid complex. It provides vouchered molecular references to support future taxonomic and ecological research in urban ecosystems.

## Simple Summary

In this study, we used an integrative approach to investigate the complex of parasitoids associated with the jumping plant-louse *Cacopsylla pulchella*, a common sap-sucking pest of the Judas tree (*Cercis siliquastrum*) in urban areas. We identified *Prionomitus mitratus* as the main parasitoid of the pest, with two species of *Pachyneuron* acting as hyperparasitoids. By combining morphological identification with DNA analysis, we confirmed species identities and generated new genetic data for public databases. These findings enhance our understanding of the natural regulation of *C. pulchella* and support future taxonomic research on parasitoids in anthropogenic areas.

## 1. Introduction

The jumping plant-louse *Cacopsylla pulchella* (Löw 1877) (*Hemiptera*: *Psyllidae*) is a sap-sucking pest likely native to the Eastern Mediterranean basin and Asia Minor [1]. Although it was first officially recorded in France in 1964 [2], historical accounts by Targioni Tozzetti [3] suggest its presence in Italy as early as the end of the 19th century. In that period, the psyllid was mistakenly reported as a “*Typhlociba*” species (Cicadellidae) infesting *Cercis siliquastrum* L. 1753. However, considering that no other specific pest of *C. siliquastrum* belonging to either Auchenorrhyncha or Psylloidea was known in Europe at that time, this record is here interpreted as a likely early misidentification of *C. pulchella*. The psyllid is now established throughout Europe [4,5,6,7,8,9,10,11,12,13,14,15,16,17,18,19].

The sap-sucking pest is monophagous and develops on *C. siliquastrum*, commonly known as the Judas tree, which is typically found in urban and peri-urban areas as an ornamental plant [10], but has also been found on *C. canadensis* Linnaeus 1753 [16]. All its stages are found on leaves of the host, mostly on their undersides. Nymphal feeding causes chlorosis and wilting of leaves and promotes the formation of sooty molds on the abundant honeydew secretions. Damage reduces photosynthetic activity, harming host tree health and reducing their ornamental value [8]. It has been documented that species from the genus *Cacopsylla* Ossiannilsson 1970 are carriers of phytoplasma disease [20], but the spread of diseases transmitted by *C. pulchella* as a vector is still to be studied [21]. The voltinism of the species depends on the number of spring flushes of the Judas tree. In Europe, *C. pulchella* typically completes three generations per year in Italy and Russia [8,16], two in France [22], and one in Switzerland and one in Serbia [10,14].

Little information is available in the literature about the presence and effectiveness of natural enemies of *C. pulchella*. Among the predators, adults of several species of Coccinellidae, namely *Adalia bipunctata* (Linnaeus 1758), *Harmonia axyridis* (Pallas 1773), *Propylea quatuordecimpunctata* (Linnaeus 1758), and *Oenopia (=Synharmonia)* (Linnaeus 1758), were reported in colonies of *C. pulchella* nymphs [8,16]. Additionally, *Wesmaelius subnebulosus* (Stephens 1836) (Neuroptera: Hemerobiidae) and several Syrphidae (Diptera) larvae have been observed [8]. More recently, predation activity by *Anthocoris nemoralis* (Fabricius 1794) (*Hemiptera*: Anthocoridae) was detected in France and Spain [23,24], while eggs of *Chrysoperla carnea* (Stephens 1836) (Neuroptera: Chrysopidae) have been observed only rarely [24].

As reported in Universal Chalcidoidea Database [25], three parasitoid species belonging to the family *Encyrtidae* (Hymenoptera) have been associated with the jumping plant-louse: *Psyllaephagus provincialis* Panis and Onillon 2013 in France [22], *Prionomitus mitratus* (Dalman 1820) and *Copidosoma breviclava* Hoffer in Greece 1970 [26]. Onillon [24] reported that parasitism on *C. pulchella* was dominated by the endoparasitoid *Psyllaephagus provincialis* (*Encyrtidae*), with lesser contributions from *Prionomitus* sp. (*Encyrtidae*) and *Pachyneuron* sp. (*Pteromalidae*), while *Tamarixia* sp. (Eulophidae) occurred as a minor ectoparasitoid. An unidentified *Psyllaephagus* species was also recorded in Tuscany in 1996 [8]; however, no study has been conducted in Italy on the biocoenosis of natural enemies of the psyllid since then. For the genus *Pachyneuron*, species-level identification based on morphological characters is particularly challenging, as evidenced by numerous reports in the scientific literature in which taxonomic determinations frequently remain at the genus level. Furthermore, in this case, associations with the host are difficult to document, and some hyperparasitic wasps were initially considered primary parasitoids. To fill this gap, observations were conducted on the collected material to clarify the trophic relationships of primary and secondary parasitoids associated with the psyllid. Additionally, we conducted an integrative study combining morphological analysis and DNA barcoding of the parasitoids associated with *C. pulchella* collected in central and southern Italy between 2022 and 2025. In several cases, these analyses have been deposited in the specialized database for the first time. The results revealed that *P. mitratus* is the primary parasitoid of the jumping plant-louse. The *Encyrtidae* are, in turn, subject to secondary parasitization by two species of *Pteromalidae* of the genus *Pachyneuron* Walker, namely *Pachyneuron muscarum* (Linnaeus 1758) and *Pachyneuron aphidis* (Bouché 1834), and one eupelmid of the genus *Anastatus* Motschulsky 1859. By linking morphological and molecular data, this work helps resolve ambiguities in existing GenBank entries and provides reliable reference sequences for future research on chalcidoid wasps.

## 2. Materials and Methods

### 2.1. Field Collections

Between July 2022 and August 2025, samplings were conducted at 12 sites in two regions: Campania (southern Italy) and Marche (central Italy) (Table 1). The inspections focused on *C. siliquastrum* plants in private gardens and public parks or used as urban trees. For each sampling, about 20 fully developed leaves infested by the jumping plant-louse were randomly collected, stored in the aerated plastic boxes and transferred to the laboratory for in-depth observations. Each infested leaf was examined within 24 or 48 h of sample collection under a stereo microscope (Leica MZ16, Leica, Wetzlar, Germany). Parasitized nymphs of the psyllid were detached from the leaves and stored individually in natural gelatin capsules (13.59 mm × 5.57 mm), at 25 ± 2 °C, 65 ± 10% relative humidity and 16:8 (L:D) photoperiod. Observations also included signs of hyperparasitism and identifying the primary host species in which secondary parasitization occurred. Consequently, for each sampling, a few parasitized nymphs were dissected to detect the presence of hyperparasitoids. Emerged adult parasitoids were killed in 70% ethanol and stored at −20 °C until morphological and molecular analyses.

### 2.2. Morphological Analysis

Adult parasitoid specimens used for taxonomic analysis were dry mounted or slide-mounted in Canada balsam–phenol medium [27]. The specimens were examined and, when required, photographed under the microscope (Leica DMLS, Leica, Wetzlar, Germany). Dichotomous keys from the literature were used to identify parasitoids [28,29,30,31,32,33,34,35,36,37,38]. The specimens were also compared with those deposited at the “Filippo Silvestri Museum” of the Department of Agricultural Sciences, University of Naples Federico II, Portici, Italy.

### 2.3. Molecular Methods for Parasitoid Identification

Molecular analyses were conducted to provide species-level validation of morphologically characterized *P. mitratus* and its *Pachyneuron* spp. hyperparasitoids, supporting integrative taxonomy.

Newly emerged wasps collected from surveys listed in Table 1 and selected based on morphological characteristics were individually preserved in absolute ethanol and stored at −20 °C until DNA processing. Before extraction, parasitoids were surface-sterilized (1% Tween-20 solution for 1 min; 1% NaOCl for 1 min; three rinses in sterile ddH2O) to remove external contaminants. Genomic DNA was obtained non-destructively using the DNeasy Blood and Tissue Kit (QIAGEN, Hilden, Germany), according to the manufacturer’s guidelines except for the initial digestion step, in which whole specimens were incubated overnight at 56 °C in ATL buffer with 10 µL of proteinase K [39]. After extraction, the specimens were retained in 70% ethanol and used for the subsequent morphological analysis. DNA yield and purity were assessed spectrophotometrically using a NanoDrop system (Thermo Fisher Scientific, Ferentino (FR), Italy) by measuring the 260/280 absorbance ratio. The 5′ region of the mitochondrial gene encoding cytochrome oxidase subunit I (COI) and the D2 expansion region of 28S rDNA were selected as molecular markers and amplified using primer pairs LCO1490/HCO2198 [40] and D2-3566F/D2-4068R [41], respectively. PCR reactions were performed in 25 µL volumes containing DreamTaq Green 2× PCR Master Mix (Thermo Fisher Scientific), 12.5 µL master mix, 0.25 µM of each primer, 0.6–2 µL template DNA and ultrapure water to volume, with thermocycling conditions following Cerasa et al. [42]. PCR products were checked on 1.2% agarose gels stained with SYBR™ Safe (Thermo Fisher Scientific) and visualized using a Chemidoc (Bio-Rad, Segrate (MI), Italy) system, after which successful amplicons were purified and sequenced bidirectionally by Eurofins Genomics (Ebersberg, Germany). Chromatograms were inspected “by eye” in BioEdit v.7.2.5 [43], trimmed for low-quality bases and assembled into consensus sequences. For COI, open reading frames were validated via translation using EMBOSS Transeq to detect stop codons or frameshifts. Final contigs were aligned with ClustalW in MUSCLE v.3.8, examined for genetic variation, queried against the reference databases with the online BLASTn (https://blast.ncbi.nlm.nih.gov/Blast.cgi, accessed on 13 November 2025) tool, and subsequently submitted to GenBank.

## 3. Results

### 3.1. Morphological Analysis

**Figure 1:**
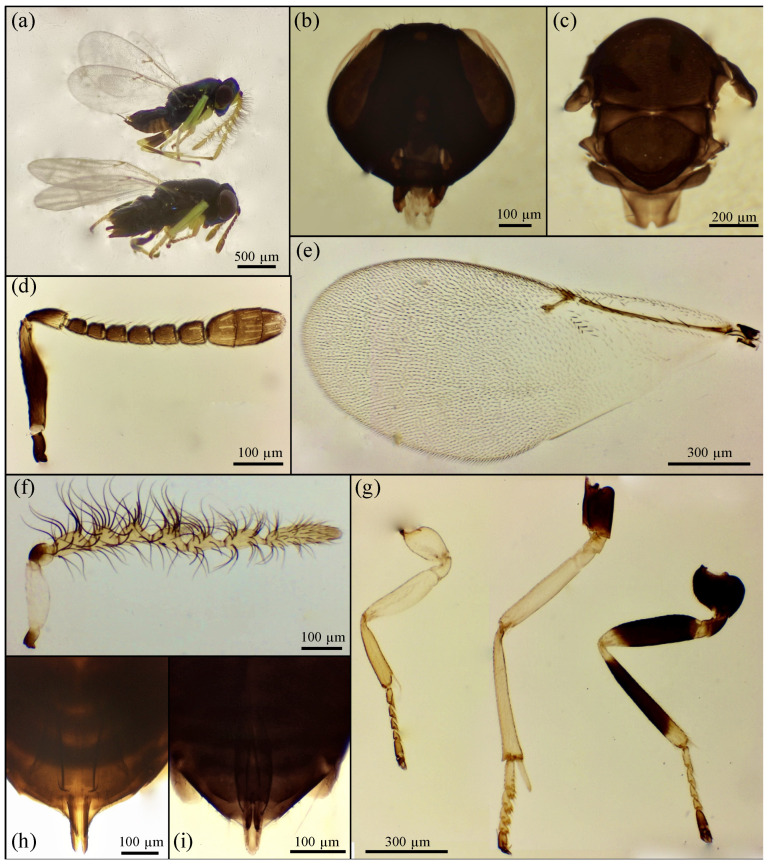
*Prionomitus mitratus*: (**a**) ♀ (down) and ♂ (up); (**b**) ♀ head; (**c**) ♀ thorax; (**d**) ♀ antenna; (**e**) ♀ forewing; (**f**) ♂ antenna; (**g**) ♀ fore, middle and hind legs (starting from the left); (**h**) ♀ gonapophyses, hypopygium and apex of the gaster; (**i**) ♂ gonapophyses.

**Figure 2:**
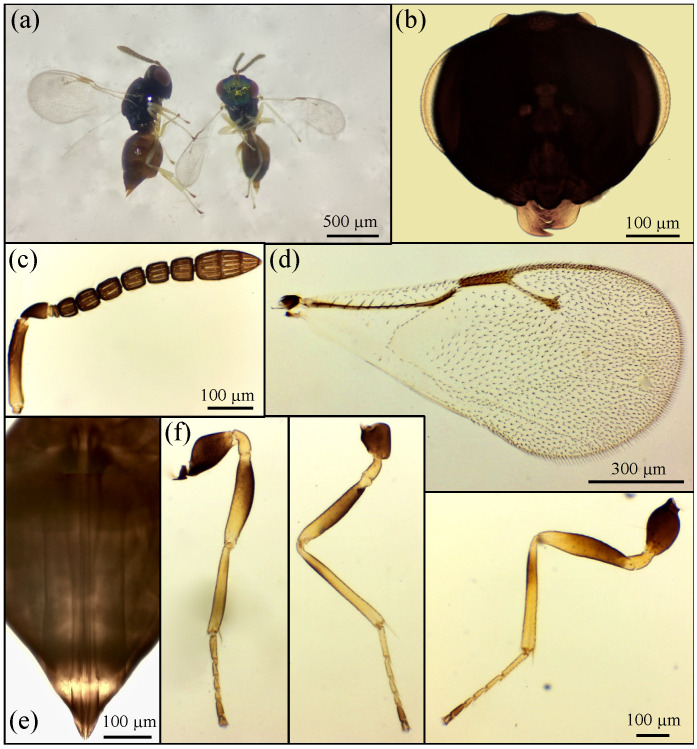
*Pachyneuron muscarum*: (**a**) ♀ (**left**) and ♂ (**right**); (**b**) ♀ head; (**c**) ♀ antenna; (**d**) ♀ forewing; (**e**) ♀ ovipositor; (**f**) ♀ fore, middle and hind legs (starting from the left).

**Figure 3:**
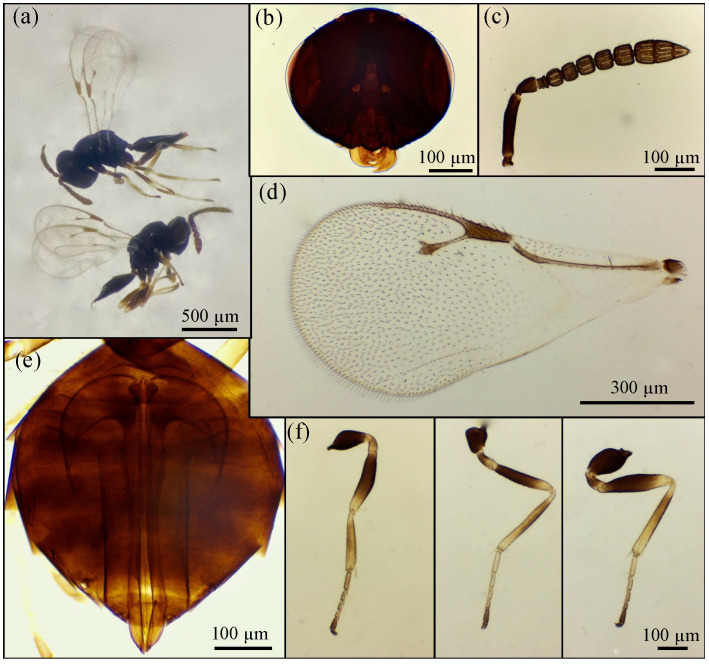
*Pachyneuron aphidis*: (**a**) ♀ (up) and ♂ (down); (**b**) ♀ head; (**c**) ♀ antenna; (**d**) ♀ forewing; (**e**) ♀ ovipositor; (**f**) ♀ fore, middle and hind legs (starting from the left).

**Figure 4:**
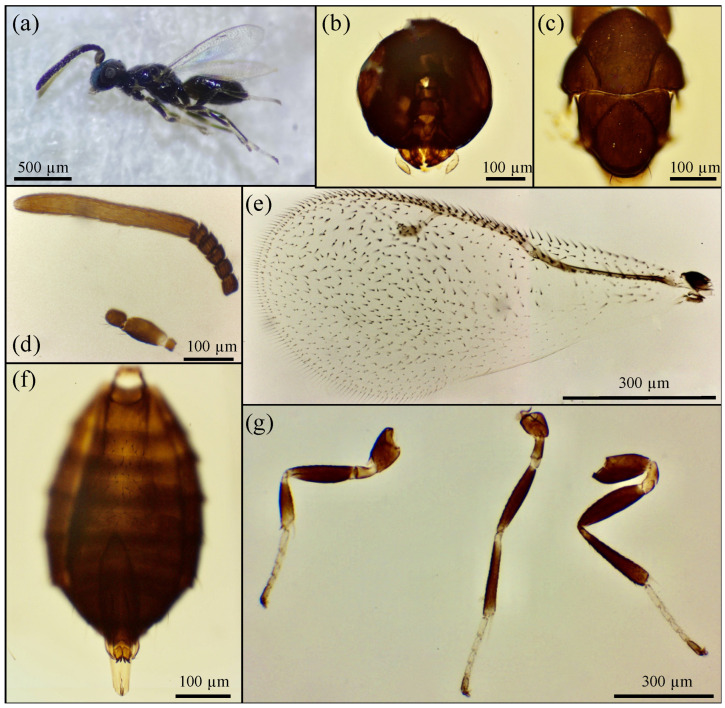
*Anastatus bifasciatus*: (**a**) male; (**b**) ♂ head; (**c**) ♂ thorax; (**d**) ♂ antenna; (**e**) ♂ forewing; (**f**) ventral view of gaster and genitalia; (**g**) ♂ fore, middle and hind legs (starting from the left).

Based on the morphological features, the emerged parasitoid species were identified as *Prionomitus mitratus* (Hymenoptera: *Encyrtidae*) (Figure 1), *Pachyneuron muscarum* (Hymenoptera: *Pteromalidae*) (Figure 2), *P. aphidis* (Hymenoptera: *Pteromalidae*) (Figure 3) and a male of *Anastatus bifasciatus* (Geoffroy 1785) (Hymenoptera: Eupelmidae) (Figure 4). Through dissections of parasitized specimens, we were able to ascertain that *P. mitratus* developed as the primary parasitoid on *C. pulchella* nymphs, while the two *Pteromalidae* and the Eupelmidae acted as hyperparasitoids. The number of emerged parasitoids per site and the collection data are reported in Table 2. A summary of the morphological traits relevant to the taxonomy of the species under study is provided below.

Females of *P. mitratus* exhibit a dark metallic sheen with blue-green to violet reflections (Figure 1a). The antennae (Figure 1d) consist of six funicular segments and a three-segmented club. The scape, black with metallic reflections except for its yellow tip, is approximately three times as long as it is wide. The pedicel, yellow ventrally and darker dorsally and basally, equals the combined length of the first two funicular segments. Funicular and club segments are yellow-brown; the first two are nearly square, while the remaining segments progressively exceed their width in length. The last segment is less than twice as long as wide, and the club equals the length of the last three segments combined.

The forewing (Figure 1e) is hyaline and approximately 2.3 times longer than wide, with a marginal vein ending in a pointed apex. The forelegs, including the coxae, are entirely yellow; the middle legs are yellow with dark coxae; the hind legs bear black femora and tibiae medially (Figure 1g). Males resemble females except for the antennae (Figure 1f). Gonapophysis, hypopygium, and apex of the gaster of both sexes (Figure 1h,i).

Female *P. muscarum* exhibits a bluish-black to dark blue body. The head is characterized by a strongly protruding lower margin of the clypeus with a convex surface and an apical margin rounded medially (Figure 2b). The antennal formula is 1-1-2-6-3 (Figure 2c). A speculum characterizes the forewing closed posteriorly. The marginal vein is as long as the stigmal vein (Figure 2d). The legs with dark coxae, femora darkened medially, and tibiae, especially the hind ones, dark, while trochanters and tarsi are yellowish except for the apical segments, which are darkened (Figure 2f).

Males differ by the bright green coloration of the head and thorax, which remains noticeable on the forehead even in specimens with a predominantly dark blue body (Figure 2a).

*P. aphidis* females are characterized by a dark brown body with variable-intensity metallic green reflections. The head bears a clypeus with a strongly produced anterior margin, rounded medially (Figure 3b). The antennal formula is 1-1-3-5-3 (Figure 3c). The forewing is characterized by a speculum open posteriorly and a marginal vein measuring 2.75–3 times its width (Figure 3d). The legs have dark coxae and femora, yellowish towards the apices; tibiae are dark, while trochanters and tarsi are yellowish, with apical segments darkened (Figure 3f).

Males resemble females except for the antennal formula, which is 1-1-2-6-3.

Since only a male of *A. bifasciatus* was collected (Figure 4a), its main diagnostic features, unique within the genus, are shown in Figure 4 and summarized below. The antenna has 5 segments on the funicle and a complete club. The latter is very developed, more than twice the length of all the flagellum segments (Figure 4d). The forewing has the costal cell dorsally with setae along the entire margin and a large quadrangular speculum (Figure 4e). The legs are mostly dark, with light spots on the trochanters, on the tips of the tibiae, and on the basal segments of the tarsi (Figure 4g).

### 3.2. Molecular Analysis

Mitochondrial and nuclear sequences were successfully generated for all examined parasitoids and subsequently deposited in GenBank (Table 3). This dataset includes the first molecular records for *P. mitratus*, for which no sequence data were previously available.

For specimens morphologically identified as *P. muscarum*, COI BLAST analysis revealed high congruence with a conspecific reference sequence (GenBank: KY912693.1; 99.53% identity), whereas the corresponding 28S-D2 sequences showed their closest affinity to a record identified only at the family level as *Pteromalidae* sp. (GenBank: KU499429.1; 99.64%).

In contrast, *Pa. aphidis* produced a more complex COI BLASTn profile. The highest-scoring match was a sequence attributed to *P. solitarium* (GenBank: KY912696.1; 99.17% identity, 92% query coverage), followed by several closely related accessions identified only at the family level, with lower similarity values (95.60–95.98%). Conversely, the corresponding nuclear sequence matched *P. aphidis* (GenBank: PP493899.1), as well as additional *Pachyneuron*/*Pteromalidae* entries, with complete identity.

## 4. Discussion

In parasitic Hymenoptera, integrative taxonomic approaches have become increasingly important for accurate species identification [44,45,46]. This is especially true for parasitoid-hyperparasitoid systems, where both primary and secondary associations belong to groups that remain poorly studied.

In this study, we combined morphological and molecular data to characterize the parasitoid complex of the jumping-plant louse *C. pulchella* in central-southern Italy, confirming the identity of *P. mitratus* as the primary parasitoid and documenting its *Pachyneuron* hyperparasitoids.

The genus *Prionomitus* Mayr 1876 comprises 9 species worldwide, 7 of which occur in the Palearctic region [38]. Host associations have been documented primarily for *P. mitratus* and *P. tiliaris* (Dalman 1820), both parasitoids of psyllid nymphs from several genera, including *Cacopsylla*; *Psylla* Geoffroy 1762; *Acizzia* Heslop-Harrison 1961; *Livilla* Curtis 1836; *Euglyptoneura* Heslop-Harrison 1961 (=*Caenothia* Heslop-Harrison 1961); *Pexopsylla* Jensen 1957; *Purshivora* Heslop-Harrison 1961 and *Nyctiphalerus* Bliven 1955 [25,38,47,48,49,50]. Additional hosts such as *Macrohomotoma gladiata* Kuwayama 1908 (*Hemiptera*: Carsidaridae) and *Agonoscena pistaciae* Burckhardt and Lauterer 1989 (*Hemiptera*: Aphalaridae) have also been reported as parasitized by *P. mitratus* [50].

According to Noyes [38], the genus *Prionomitus* is morphologically related to *Psyllaephagus*, from which it can be distinguished by the female hypopygium, which extends to the apex of the gaster, enclosing the ovipositor almost to its apex (Figure 1a,h). In *Psyllaephagus*, on the other hand, the hypopygium is visibly shorter and does not reach the apex of the gaster [38]. In addition, specimens of both sexes in the genus *Prionomitus* are characterized by the presence of a posterolateral depression on the mesoscutum anterior to each tegula (Figure 1c), which is absent in *Psyllaephagus* [31,38].

The type species of *P. mitratus* was first described by Dalman [51] under the names *Encyrtus mitratus* and *Encyrtus chlorinus*. Mayr [52] defined the genus *Prionomitus*, a definition later adopted by all subsequent authors, particularly Ferrière [53,54]. *Prionomitus mitratus* is a Holarctic species [25], commonly associated with pear and hawthorn psyllids in Europe and North America. The parasitoid usually attacks 4th and 5th instar nymphs on which it develops as a primary parasitoid [47,50,53,55,56,57,58,59,60,61,62,63]. Given its importance as a parasitoid of pear psyllids in France, Delvare et al. [64] redescribed it to clarify its morphology and biology.

A total of 8 females and 11 males of *P. mitratus* have been collected from *C. pulchella* in 7 of the 12 monitored sites. The encyrtid emerged only from samples collected between March and early June. The only exception concerns the collections carried out in San Ginesio (MC), where the primary parasitoid was collected in late August. According to our results, *P. mitratus* was obtained from the same host in Greece [26].

*Pachyneuron* Walker is a cosmopolitan genus with about 64 valid species [25]. Most species develop on primary parasitoids of plant-sucking *Hemiptera* (Aphidoidea, Coccoidea, Psylloidea), among them the different species of Braconidae (Hymenoptera: Ichneumonoidea), Aphelinidae and *Encyrtidae* (Hymenoptera: Chalcidoidea) [33]. The genus *Pachyneuron* is also associated, as a primary or secondary parasitoid, with aphidiphagous Diptera, in particular Syrphidae and Chamaemyidae; Chrysopidae; the larvae of Coccinellidae; and the eggs of Lepidoptera [33,35].

The pteromalid *P. muscarum* was originally described by Linnaeus under the name *Ichneumon muscarum* L. based on the description previously provided by De Geer. After many years of confusion about the taxonomic status of this species, Boućek proposed its definitive combination [65]. In our collections, *P. muscarum* was the most abundant hyperparasitoid, with 49 males and 15 females. It was recorded at most of the sites investigated, with a prevalence in Casagiove (CE). In a few locations, it was not found, likely due to the rarefaction of the host. The pteromalid is a cosmopolitan species, extremely polyphagous and is commonly collected from Coccoidea, Aphidoidea, Psylloidea and Cucujoidea as a secondary parasitoid of *Encyrtidae*, Braconidae, Aphidiinae, and Eulophidae [28,34,35,66]. The characteristics related to the biological development and behavior of the pteromalid hyperparasitoid have been studied by rearing *P. muscarum* on *Microterys flavus* (Howard) (Hymenoptera: *Encyrtidae*), which was left to develop as a primary parasitoid on *Coccus hesperidum* L. [67]. Moreover, the same authors demonstrated that *P. muscarum* can develop as a primary parasitoid of the pupae of aphidophagous predators, such as Syrphidae [68]. According to our results, Novak [47] reported *P. muscarum* as the dominant hyperparasitoid of hawthorn psyllids through *P. mitratus* and *P. tiliaris*. Similarly, it was reported on *C. pyri* (Linneus 1758) and *C. pyrisuga* (Förster 1848) through *Tamarixia psyllae* Yefremova and Yegorenkova 2009, and *P. mitratus* [57,58,60,61,63,69].

*Pachyneuron aphidis* is a cosmopolitan species primarily known to act as a hyperparasite of various aphids [70]. This species was described as *Diplolepis aphidis* Bouché in 1834, then transferred to the genus *Pachyneuron* by Reinhard [71], which was accepted by Graham [28]. Its host range is wide and includes numerous families belonging to *Hemiptera*, including *Psyllidae* [25,33,35,36,72]. In the literature, associations with species of Coccinellidae, Agromyzidae, Cecidomyiidae, Gelechiidae and Tortricidae are also reported [25,33,36,66]. In our surveys, with a total of 3 males and 7 females, it resulted in being the second most common hyperparasitoid species, following *P. muscarum*. Overall, it was mostly reared in San Giorgio a Cremano (NA). Considering that the previous records of *P. aphidis* from psyllids regarded the emergence from *C. pyri*, *C. pyrisuga* and from an unidentified *Cacopsylla* [25,61,69,73,74], our finding represents its first association with *C. pulchella*.

Taken together, our findings suggest that the hyperparasitic activity of *Pachyneuron* spp. is most pronounced during the summer months. Recent studies on how congeneric parasitoids share hosts indicate that coexistence can emerge through multiple ecological axes, most often seasonal changes in activity, distinct preferences for host/primary parasitoid stage, differences in microhabitat, and divergent oviposition tactics [75]. In our system, the summer peak of *Pachyneuron* suggests a partial temporal offset between *P. muscarum* and *P. aphidis*, consistent with the natural framework described in the literature.

The emergence of only one male of *A. bifasciatus* from a *C. pulchella* nymph in San Ginesio (MC) represents an unusual finding. The species of *Anastatus* are mostly primary endoparasitoids of a wide diversity of insect eggs, though some have been reared as hyperparasitoids [30]. Viggiani and Tremblay [76] obtained a female of *A. bifasciatus* from the aphid *Cinara schimitscheki* Boer. parasitized by *Pauesia pini* (Hal.) (Braconidae: Aphidiinae). The same authors assumed that the parasitization of hosts in a systematic manner is a phenomenon of morphotypic specialization. In the latter case, parasitoids may attack a wide range of hosts sharing similar external appearances [76,77]. Similarly, in our case, the emergence of the eupelmid could be linked to a rarefaction of parasitizable hosts in the monitored area, which would then have determined, in the absence of alternatives, the parasitization of the psyllid nymph. In addition, Trjapitzin [78] mentioned *A. bifasciatus* as a secondary parasite of *C. pruni* (Scopoli 1763) and *C. pyri*, but the source of this finding is not reported by the author. For this reason, future studies could be useful to verify the recurrence of *A. bifasciatus* on psyllid nymphs, as well as its trophic role and the simultaneous presence on other hosts in the same area.

To complement the morphological study, we applied a dual-marker barcoding approach to confirm the identity of *P. mitratus* and its *Pachyneuron* hyperparasitoids, selecting the COI and 28S-D2 regions for their widespread use in Chalcidoidea systematics [44,79]. The newly generated sequences for *P. mitratus* represent the first molecular reference for this encyrtid, thereby improving its taxonomic documentation and enabling future comparative and phylogenetic works.

The molecular data obtained in this study further corroborate the morphological identification of *P. muscarum*. The COI barcode showed full concordance with an existing conspecific reference, whereas the corresponding nuclear sequence matched only at the family level. Notably, in the study from which the closest 28S-D2 reference originates [80], the authors reported that a subset of specimens could not be confidently assigned to species and were likely *Pachyneuron* hyperparasitoids, thereby highlighting the taxonomic gaps that persist within this group.

For *P. aphidis*, the nuclear marker supported morphological identification, while the COI sequence showed greatest similarity to *P. solitarium* and to unnamed *Pteromalidae* accessions. In any case, the two species are easily distinguishable morphologically based on the antennal formula, indicating that species-level mitochondrial references for this taxon remain poorly represented. Such outcomes are expected in large public repositories, where reference coverage and annotation quality can vary markedly among taxa [81,82]. Consequently, concordance between morphology and an independent nuclear marker remains critical for species attribution in *Pachyneuron*.

By providing vouchered, morphologically validated sequences for all three parasitoid species, this study expands the molecular resources for parasitoids associated with *C. pulchella*. The integrative approach adopted here demonstrates how combining morphology with genetic evidence can enhance taxonomic resolution in chalcidoid Hymenoptera.

## 5. Conclusions

This study documents the first integrative characterization of the parasitoid complex associated with *Cacopsylla pulchella* on *Cercis siliquastrum* in urban areas of central and southern Italy. By combining diagnostic morphology with DNA barcoding, we confirmed *Prionomitus mitratus* as the primary parasitoid and identified *Pachyneuron muscarum*, *P. aphidis*, and a single specimen of *Anastatus bifasciatus* as hyperparasitoids. Beyond clarifying the trophic structure of this host-parasitoid network, the vouchered molecular dataset generated here expands the reference genetic sequences required for future comparative, phylogenetic and population-level investigations. Lastly, further work based on quantitative and standardized sampling designs will be essential to assess the parasitism and hyperparasitism rates, estimate seasonal and spatial variation, and better quantify the biological control services provided by these parasitic wasps in shaping psyllid population dynamics in urban environments

## Figures and Tables

**Table 1:** Sampling localities in Southern and Central Italy. (NA): Metropolitan city of Naples, Campania region; (CE): Province of Caserta, Campania region; (SA): Province of Salerno, Campania region; (AN): Province of Ancona, Marche region; (MC): Province of Macerata, Marche region.

Localities	Coordinates	Data of the Sampling
San Giorgio a Cremano (NA)	40.825667 N, 14.329694 E	23 July 2022
		24 March 2023
		3 July 2023
		25 March 2024
Caserta (CE)	41.075761 N, 14.336000 E	25 July 2023
		25 May 2024
		15 June 2025
Napoli (NA)	40.845111 N, 14.257879 E	14 April 2024
	40.834528 N, 14.313944 E	20 July 2025
Casagiove (CE)	41.076687 N, 14.309078 E	29 May 2024
		10 May 2025
Loreto (AN)	43.445018 N, 13.617268 E	2 June 2024
San Sebastiano al Vesuvio (NA)	40.841522 N, 14.369364 E	20 July 2024
Vallo della Lucania (SA)	40.231562 N, 15.266000 E	21 July 2024
Palma Campania (NA)	40.861417 N, 14.546111 E	25 July 2024
Portici (NA)	40.816833 N, 14.347861 E	10 May 2025
San Ginesio (MC)	43.108218 N, 13.314975 E	21 August 2025
Montecassiano (MC)	43.364354 N, 13.434405 E	29 August 2025

**Table 2:** Parasitoids emerged (♂ male; ♀ female) from psyllid nymphs in all the sampling localities in Southern and Central Italy. (NA): Metropolitan city of Naples, Campania region; (CE): Province of Caserta, Campania region; (SA): Province of Salerno, Campania region; (AN): Province of Ancona, Marche region; (MC): Province of Macerata, Marche region.

Localities	Sampling Data	Emerged Parasitoids
San Giorgio a Cremano (NA)	23 July 2022	1 ♂ + 3 ♀ *P. aphidis*; 3 ♂ *P. muscarum*
	24 March 2023	2 ♀ *P. aphidis*
	3 July 2023	6 ♂ + 1 ♀ *P. muscarum*
	25 March 2024	1 ♂ + 1 ♀ *P. aphidis*; 1 ♂ + 3 ♀ *P. muscarum*; 4 ♂ *P. mitratus*
Caserta (CE)	25 July 2023	1 ♀ *P. aphidis*; 3 ♂ + 1 ♀ *P. muscarum*
	25 May 2024	2 ♂ + 2 ♀ *P. mitratus*
	15 June 2025	2 ♂ + 1 ♀ *P. mitratus*
Napoli (NA)	14 April 2024	1 ♂ *P. muscarum*; 1 ♂ *P. mitratus*
	20 July 2025	2 ♂ *P. muscarum*
Casagiove (CE)	29 May 2024	12 ♂ + 7 ♀ *P. muscarum*; 1 ♀ *P. mitratus*
	10 May 2025	16 ♂ + 3 ♀ *P. muscarum*; 1 ♀ *P. mitratus*
Loreto (AN)	2 June 2024	1 ♀ *P. mitratus*
San Sebastiano al Vesuvio (NA)	20 July 2024	1 ♂ *P. aphidis*
Vallo della Lucania (SA)	21 July 2024	1 ♂ *P. muscarum*
Palma Campania (NA)	25 July 2024	2 ♂ *P. muscarum*
Portici (NA)	10 May 2025	1 ♂ *P. muscarum*
San Ginesio (MC)	21 August 2025	1 ♂ *A. bifasciatus*
Montecassiano (MC)	29 August 2025	1 ♂ *P. muscarum*; 1 ♂ + 2 ♀ *P. mitratus*

**Table 3:** GenBank accession numbers for COI and 28S-D2 sequences obtained in this study, with corresponding amplicon lengths (bp).

Species	COI	28S-D2
*Prionomitus mitratus*	PX644762 (649)	PX629151 (601)
*Pachyneuron muscarum*	PX622629 (679)	PX622923 (577)
*Pachyneuron aphidis*	PX622627 (657)	PX622636 (588)

## Data Availability

The data presented in this study are openly available in [GenBank accession numbers for 28S-D2] [https://www.ncbi.nlm.nih.gov/nuccore/3121074159; 28 November 2025] [PX629151.1].
